# Sex differences in Alzheimer’s-related Tau biomarkers and a mediating effect of testosterone

**DOI:** 10.1186/s13293-020-00310-x

**Published:** 2020-06-19

**Authors:** Erin E. Sundermann, Matthew S. Panizzon, Xu Chen, Murray Andrews, Douglas Galasko, Sarah J. Banks, for the Alzheimer’s Disease Neuroimaging Initiative

**Affiliations:** 1 Department of Psychiatry, University of California, San Diego, 9500 Gilman Dr., La Jolla, CA 92093 USA; 2 Department of Neuroscience, University of California, San Diego, 9500 Gilman Dr, La Jolla, CA 92093 USA

**Keywords:** Alzheimer’s disease, Phosphorylated-Tau, Testosterone, APOE, Sex, Cerebrospinal fluid

## Abstract

Women show greater pathological Tau biomarkers than men along the Alzheimer’s disease (AD) continuum, particularly among apolipoprotein ε-E4 (APOE4) carriers; however, the reason for this sex difference in unknown. Sex differences often indicate an underlying role of sex hormones. We examined whether testosterone levels might influence this sex difference and the modifying role of APOE4 status. Analyses included 172 participants (25 cognitively normal, 97 mild cognitive impairment, 50 AD participants) from the Alzheimer’s Disease Neuroimaging Initiative (34% female, 54% APOE4 carriers, aged 55–90). We examined the separate and interactive effects of plasma testosterone levels and APOE4 on cerebrospinal fluid phosphorylated-tau181 (p-Tau) levels in the overall sample and the sex difference in p-Tau levels before and after adjusting for testosterone. A significant APOE4-by-testosterone interaction revealed that lower testosterone levels related to higher p-Tau levels among APOE4 carriers regardless of sex. As expected, women had higher p-Tau levels than men among APOE4 carriers only, yet this difference was eliminated upon adjustment for testosterone. Results suggest that testosterone is protective against p-Tau particularly among APOE4 carriers. The lower testosterone levels that typically characterize women may predispose them to pathological Tau, particularly among female APOE4 carriers.

## Background

There are critical gaps in our understanding of sex differences in Alzheimer’s disease (AD) including the higher prevalence of AD [1, 2], the steeper cognitive decline [3–5], and a stronger effect of the apolipoprotein E ε4 allele (APOE4) on AD risk in women versus men [6–8]. Sex differences in underlying AD pathology have also been reported, with autopsy [9], neuroimaging [10], and cerebrospinal fluid (CSF) [11, 12] studies reporting higher levels of pathological tau (referred here simply as “Tau”) in women versus men who are either diagnosed with or are at-risk for AD by way of the APOE4 allele or clinically significant beta-amyloid (Aβ) plaque deposition in the brain. Because Tau topography is closely tethered to clinical presentation [13], the higher levels of Tau in women may be a contributing factor to the higher prevalence and more aggressive clinical profile of AD in women.

The reasons for higher levels of Tau in women are unknown; however, a potential mechanism may stem from differences in sex hormones. Animal studies report a protective role of testosterone against the hyperphosphorylation of tau (p-Tau) in both male and female rats [14–16], suggesting that the typically lower testosterone levels in women may be a risk factor for pathological Tau. In women, circulating estradiol levels experience a substantial decline during menopause, whereas post-menopausal women continue to demonstrate a range of circulating testosterone levels that continue to be lower than levels in men [17]. Despite this sex difference in hormone levels, most studies examining links between testosterone and AD-related outcomes have been solely in men [18–23], and the link between testosterone and Tau has been minimally examined in humans.

Despite some inconsistencies [24, 25], a wealth of evidence indicates an association between low testosterone levels, poorer cognitive function [26–30], and greater odds or risk for AD [18, 20–23, 31, 32], with these associations more clearly defined in men [18, 20–23, 26–29, 31, 32] than in women [26, 27]. Suggestive of a more causative than consequential role for testosterone on AD-related outcomes, longitudinal studies have shown that low free and/or total testosterone levels precede development of AD dementia [23] and cognitive dysfunction on measures of global cognition [26, 29] and episodic memory [29]. Furthermore, exogenous testosterone supplementation led to improved performance over time in a range of cognitive domains including global cognition [19, 33], psychomotor speed [33], executive function [33], and spatial and verbal memory [34, 35], although not always [36].

Animal and human studies demonstrate that the effects of testosterone may depend on APOE genotype. The APOE4 allele is associated with lower testosterone levels in men [31], and with downregulation of androgen receptors in mice, resulting in reduced binding of testosterone [37]. Experimental manipulations of testosterone levels in male and female mouse models relate to changes in cognitive function more so among APOE4 carriers than APOE3 carriers [37, 38], suggesting that APOE4 carriers are more sensitive to the effects of testosterone on the brain. In humans, the direction of the APOE4 by testosterone interaction is less consistent, whereby Panizzon et al. found that low testosterone levels in men related to smaller hippocampal volumes and poorer episodic memory among APOE4 carriers only [39, 40], whereas Hogervorst et al. found that low testosterone levels related to a greater likelihood of an AD diagnosis among APOE4 non-carriers only [31]. The APOE4 by testosterone interaction has yet to be examined either in women or in relation to hallmark AD pathologies.

In the Alzheimer’s Disease Neuroimaging Initiative (ADNI), we aimed to replicate previous findings of greater pathological Tau biomarkers in women versus men at-risk for AD by way of the APOE4 allele, and to extend these findings by testing the hypothesis that testosterone may contribute to this sex difference. To this end, we examined the relationship of circulating total and free testosterone levels and their interaction with APOE4 with CSF levels of p-Tau across and within sex while adjusting for amyloid-β (Aβ) biomarkers and other covariates. Furthermore, we determined whether lower testosterone levels in women partially account for their higher p-Tau levels. Extrapolating from animal studies, we hypothesized that lower testosterone levels would relate to higher p-Tau levels across sex and more so among APOE4 carriers versus non-carriers. Furthermore, we hypothesized that the higher p-Tau levels in female versus male APOE4 carriers will diminish upon adjustment for testosterone. Because of a previously reported link between testosterone and Aβ pathology in a rodent model [14] and evidence of an effect of Aβ on Tau development [41], we also examined whether testosterone and Tau associations were independent of CSF Aβ levels.

## Methods

### Participants and data source

Data were extracted from ADNI, a publically accessible dataset available at adni.loni.usc.edu. ADNI is a longitudinal, multi-site, cohort study that began in 2003 as a public-private partnership. Information about ADNI can be found at www.adni-info.org. The primary goal of ADNI is to test whether neuroimaging measures and other biological and clinical markers can be combined to measure the progression of MCI and early AD. ADNI study visits involve neuroimaging, neuropsychological, and clinical and biomarker assessments. The general enrollment inclusion/exclusion criteria for ADNI have been described elsewhere [42]. This specific study was limited to ADNI1 participants with CSF p-Tau levels, as determined by the Roche Elecsys assay, and plasma testosterone levels from their baseline visit. The current sample consisted of 172 participants (113 men and 59 women) aged 55–90 years including 25 (15%) cognitively normal, 97 (56%) MCI, and 50 (29%) AD dementia individuals.

### Fluid biomarkers

Plasma levels of total testosterone and sex hormone binding globulin (SHBG) were measured on the Luminex xMAP platform by Biomarkers Consortium Plasma Proteomics Project Rules-Based Medicine multiplex (http://www.rulesbasedmedicine.com) as part of a panel of 190 analytes related to a diverse array of human disease. A Box-Cox transformation was applied to raw assay values to normalize the distribution. Detail of assay methods and normalization procedures are described in “Biomarkers Consortium Plasma Proteomics Data Primer 02Aug2013 Final.pdf” and available for download at http://adni.loni.usc.edu/data-samples/access-data/. We utilized CSF concentrations of p-Tau (pg/mL), phosphorylated at threonine 181, and Aβ as determined by the Roche Elecsys assay (Roche, Basel, Switzerland). Detailed methods and quality control procedures for p-Tau measures can be found at http://adni.loni.ucla.edu. Increased CSF p-tau_181_ levels occur in AD but not in other neurodegenerative disorders. SHBG is a protein that binds testosterone rendering it biologically unavailable. Thus, SHBG levels were used to measure levels of bound versus unbound or free testosterone using the following formula: total testosterone/SHBG × 100. All analyses were repeated substituting free for total testosterone levels to determine whether results were driven by bioavailable testosterone.

### Statistical analyses

Continuous variables that were not normally distributed were transformed via log- or Box-Cox transformations to improve normality. Sample characteristics by sex and APOE4 status were assessed using independent *t* tests for continuous variables and chi-square tests for categorical variables. First, we used linear regression to examine the separate and interactive effects of sex and APOE4 status on p-Tau levels while adjusting for age, education, and cardiovascular risk factors available in ADNI (i.e., body mass index [BMI] and self-reported history of cardiovascular events). Men were compared to women (reference group) and APOE4 carriers to APOE4 non-carriers (reference group). Next, we used linear regression to examine the effect of testosterone and its interaction with APOE4 status on p-Tau levels in the overall sample and within sex. In addition to the previously mentioned covariates, we adjusted for sex in analyses in the overall sample. Significant interactions were probed via analyses stratified by APOE4 status. Next, stepwise linear regressions were conducted in the overall sample to examine sex differences in p-Tau levels after adding testosterone (step 2) to the initial model that adjusted for age, education, and cardiovascular risk factors (step 1). Analyses were compared before and after covarying for Aβ_1-42_ levels in order to determine the specificity of findings to p-Tau.

## Results

Among 172 participants, there were 79 APOE4 non-carriers (25 women and 54 men) and 93 APOE4 carriers (34 women and 59 men). The sample was 97% White, with a mean age of 75, and mean years of education of 15. In the overall sample, APOE4 carriers were younger, showed poorer global cognition (lower mean MMSE score), had higher p-Tau levels, were less likely to be cognitively normal, and more likely to be AD dementia patients compared to non-carriers (*p*s < .05; Table 1). Mean testosterone level was lower in APOE4 carriers versus non-carriers, although not significantly (*p* = .09). When comparing men and women by APOE4 status, female APOE4 carriers were significantly younger than male APOE4 carriers (*p* = .002). As expected, mean total and free testosterone levels were lower in women than in men regardless of APOE4 status (*p*s < .001). In replication of previous findings, p-Tau levels were higher in women versus men but only among APOE4 carriers (*p* = .001).

**Table 1:** Sample characteristics by APOE4 carrier status and sex

	APOE4− (*n* = 79)	APOE4+ (*n* = 93)	*p* value (effect size)^a^	APOE4-			APOE4+		
				Women *n* = 25	Men *n* = 54	p value (effect size)^a^	Women *n* = 34	Men *n* = 59	*p* value (effect size)^a^
Age, Mean (SD)	76.6 (7.2)	74.0 (6.7)	.02 (.37)	77.0 (6.3)	76.4 (7.7)	.74	71.3 (7.4)	75.6 (5.7)	.002 (.65)
Years of education, Mean (SD)	15.9 (3.0)	15.3 (3.2)	.20	15.7 (2.7)	16.0 (3.1)	.64	14.7 (2.9)	15.6 (3.4)	.17
White, *n* (%)	76 (96.2%)	91 (97.8%)	.54	25 (100%)	51 (94.4%)	.49	32 (94.1%)	59 (100%)	.06
Cognitive status			< .001 (.40)			.26			.65
Cognitively normal, *n* (%)	22 (27.8%)	3 (3.2%)		10 (40.0%)	12 (22.2%)		1 (2.9%)	2 (3.4%)	
MCI, *n* (%)	45 (57.0%)	52 (55.9%)		12 (48.0%)	33 (61.1%)		17 (50.0%)	35 (59.3%)	
AD dementia, *n* (%)	12 (15.2%)	38 (40.9%)		3 (12.0%)	9 (16.7%)		16 (47.1%)	22 (37.3%)	
Global cognition (MMSE), Mean (SD)	27.1 (2.2)	25.8 (2.5)	< .001 (.55)	27.6 (2.2)	26.9 (2.2)	.15	25.6 (2.5)	25.9 (2.5)	.56
BMI, Mean (SD)	26.5 (4.0)	25.8 (3.7)	.28	25.7 (4.9)	26.8 (3.5)	.24	25.5 (4.0)	26.0 (3.6)	.55
Self-reported history of cardiovascular events, *n* (%)	61 (77.2%)	62 (66.7%)	.13	16 (64.0%)	45 (83.3%)	.06	23 (67.6%)	39 (66.1%)	.88
Pulse pressure^b^, Mean (SD)	61.0 (18.1)	59.1 (14.7)	.41	61.9 (23.9)	60.8 (15.0)	.80	58.9 (15.8)	59.2 (14.1)	.93
Plasma total testosterone level^c^ (ng/mL), Mean (SD)	0.2 (0.4)	0.1 (0.5)	.09	− 0.3 (0.4)	0.5 (0.1)	< .001 (2.74)	− 0.4 (0.4)	0.4 (0.2)	< .001 (2.53)
Plasma free testosterone level^c^ (ng/mL), Mean (SD)	13.4 (24.8)	6.5 (27.7)	.09	− 16.9 (19.5)	27.5 (9.8)	< .001 (2.9)	− 22.0 (22.7)	23.0 (13.0)	< .001 (2.4)
CSF p-Tau_181_ level (pg/mL), Mean (SD)	26.8 (13.1)	35.9 (17.2)	< .001 (.59)	23.5 (8.6)	28.3 (14.5)	.13	43.7 (22.6)	31.5 (11.1)	.001 (0.68)
CSF Aβ_1-42_ level (pg/mL), Mean (SD)	1240.4 (702.9)	639.1 (292.2)	< .001 (1.12)	1286.7 (765.1)	1218.9 (678.7)	.69	658.7 (627.8)	627.8 (327.0)	.63

^a^Effect sizes are provided for significant differences; Cohen’s *d* is provided for mean differences (0.2 = small, 0.5 = medium, 0.8 = large) and a phi coefficient is provided for differences in proportions (0.1 = small, 0.3 = medium, 0.5 = large)

^b^Pulse pressure = systolic − diastolic blood pressure

^c^Testosterone levels were normalized based on a Box-Cox transformation. *MCI* mild cognitive impairment, *AD* Alzheimer’s disease, *APOE4* apolipoprotein E ɛ4 allele, *MMSE* Mini Mental Status Examination, *BMI* body mass index, *CSF* cerebrospinal fluid

### Sex differences in p-Tau by APOE4 status

**Fig. 1:**
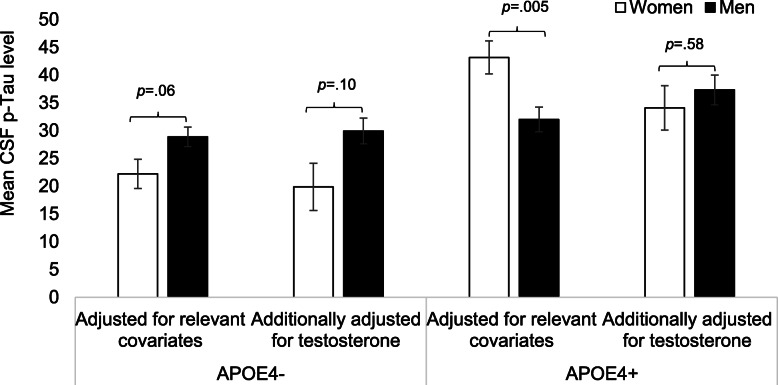
Sex differences in CSF p-Tau by APOE4 status before and after adjusting for testosterone levels. Using multiviariable linear regression, we found a significant sex by APOE4 status interaction on p-Tau levels revealing higher p-Tau levels in women versus men among APOE4 carriers (women: *n* = 34, mean = 43.16, SD = 2.97; men: *n* = 59, mean = 32.00, SD = 2.23) but not among APOE4 non-carriers (women: *n* = 25, mean = 22.61, SD = 2.65; men: *n* = 54, mean = 28.70, SD = 1.76). The significant sex difference in p-Tau levels among APOE4 carriers was eliminated when adjusting for testosterone levels in addition to relevant covariates. *CSF* cerebrospinal fluid, *p*-*Tau* phosphorylated Tau, *APOE4* apolipoprotein E ε4 allele. Relevant covariates were age, education, BMI, and self-reported history of cardiovascular events. Testosterone represents plasma-based total testosterone levels

In line with hypotheses and our unadjusted analyses (Table 1), a significant sex by APOE4 interaction on p-Tau levels (*B* = − 5.76, β = − 0.24, standard error [SD] = 2.96, *p* = .05) when adjusting for covariates (i.e., age, education, and cardiovascular risk factors) indicated higher p-Tau levels in women versus men among APOE4 carriers only (*B* = − 11.16, β = − 0.31, SD = 3.85, *p* = .005). Analyses stratified by APOE4 status actually revealed an opposing sex difference among non-carriers, whereby p-Tau levels were higher in men versus women, although not significantly (*B* = 6.09, β = 0.22, SD = 3.22, *p* = .06; Fig. 1).

### Relationship between testosterone and p-Tau by APOE4 status

**Fig. 2:**
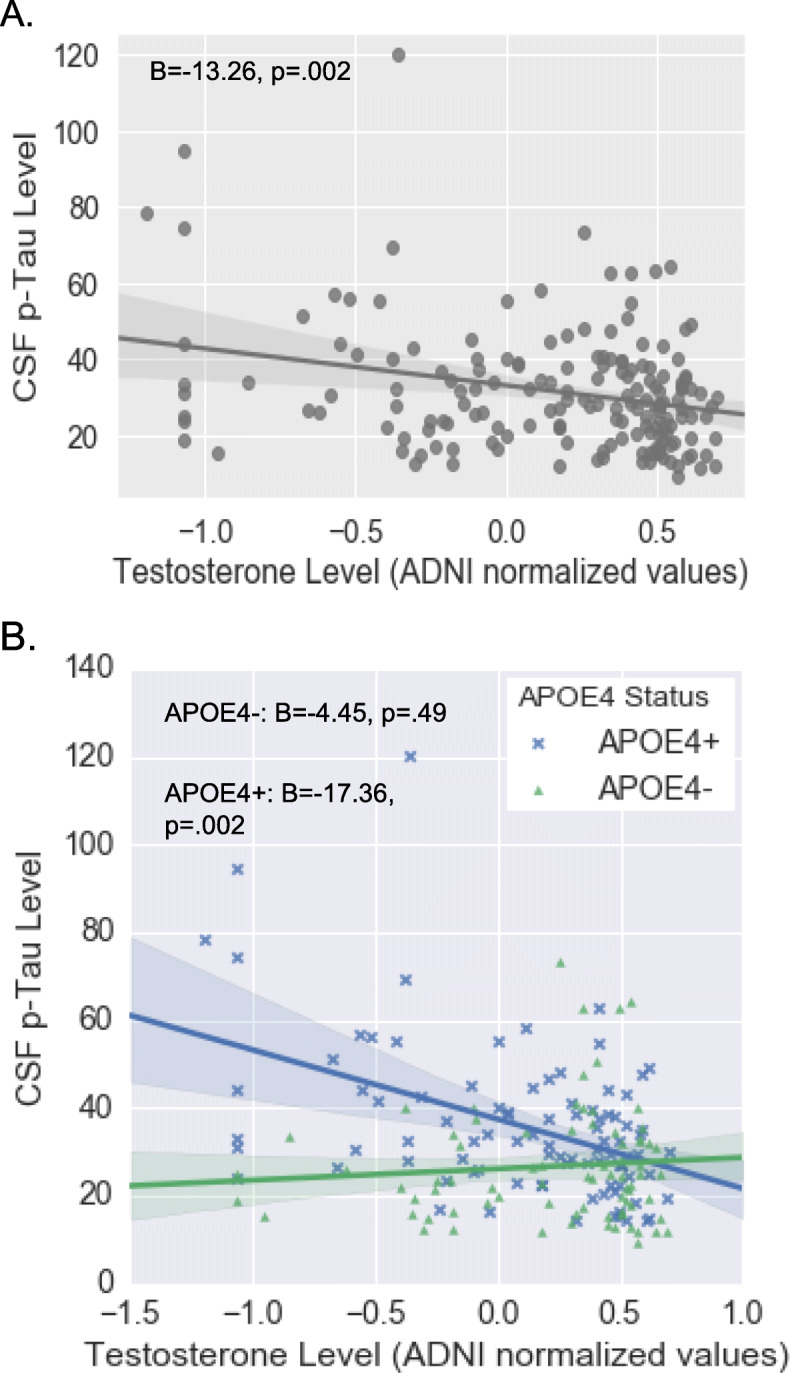
Relationship between plasma testosterone levels and CSF p-Tau levels overall (**a**) and by APOE4 status (**b**). Using multivariable linear regression, we found a significant total testosterone X APOE4 status interaction on p-Tau levels that revealed an association between lower total testosterone levels and higher p-Tau levels among APOE4 carriers (*n* = 93) but not among APOE4 non-carriers (*n* = 79). *CSF* cerebrospinal fluid, *p*-*Tau* phosphorylated Tau, *APOE4* apolipoprotein E ε4 allele. Analyses covaried for age, sex, education, BMI, and self-reported history of cardiovascular events

In the overall sample, there was a significant relationship between lower total testosterone levels and higher CSF p-Tau levels (*B* = − 13.26, *β* = − .39, *p* = .002; Fig. 2), but, more importantly, there was a significant total testosterone X APOE4 status interaction on p-Tau levels (*B* = − 17.78, β = − 0.40, SD = 4.9, *p* < .001). Analyses stratified by APOE4 status revealed that lower total testosterone levels were associated with higher p-Tau among APOE4 carriers (*B* = − 17.36, β = − 0.50, SE = 5.41, *p* = .002) but not non-carriers (*B* = − 4.45, β = − 0.15, SE = 6.4, *p* = .49). Results in the overall and the APOE4-stratified analyses were unchanged when substituting free for total testosterone and when including Aβ levels as a covariate in the model.

**Fig. 3:**
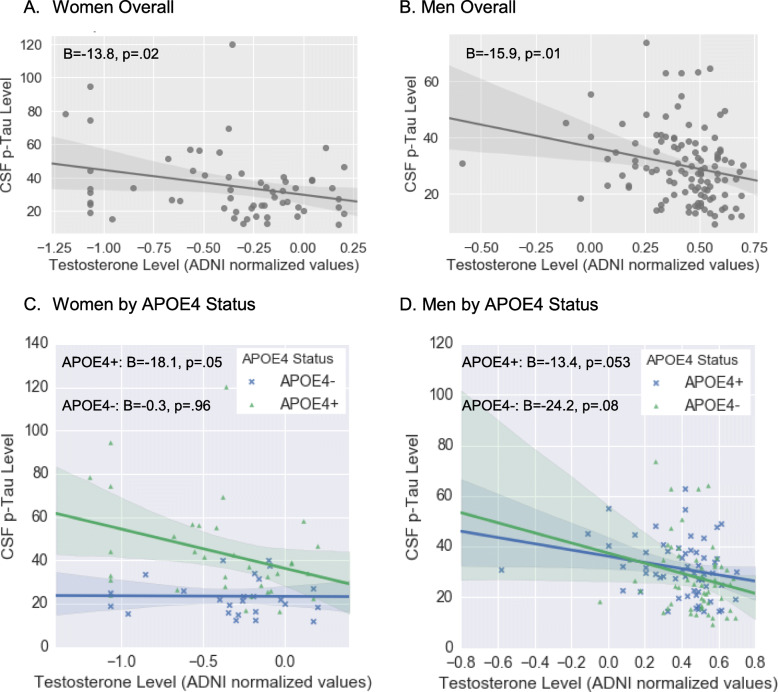
The sex-stratified relationship between plasma testosterone levels and CSF p-Tau levels overall (**a**, **b**) and by APOE4 status (**c**, **d**). Using multivariable linear regression, we found negative associations between testosterone and p-Tau levels in both women and men. Although the testosterone X APOE4 status interactions were not significant in either sex, the significant testosterone X APOE4 interaction in the overall sample appeared to be driven by women. When analyses were stratified by sex, we found that the testosterone X APOE4 status interaction on p-Tau levels was driven by women. *CSF* cerebrospinal fluid, *p*-*Tau* phosphorylated Tau, *APOE4* apolipoprotein E ε4 allele. Analyses adjusted for age, education, BMI, and self-reported history of cardiovascular events

In sex-stratified analyses, the range of total testosterone levels were lower in women (range = − 1.2–0.2, median = − 0.28) versus men (range − 0.6–0.7, median = 0.5), although overlapping. Within the distribution of lower testosterone levels in women (*B* = − 13.83, β = − 0.27, SE = 5.88, *p* = .02) and the distribution of higher levels in men (*B* = − 15.85, β = − 0.24, SE = 6.32, *p* = .01), there was a negative association between testosterone and p-Tau levels suggestive of a continuous, linear relationship (Fig. 3). These associations occurred regardless of APOE4 status as indicated by non-significant testosterone by APOE4 interactions in women (*B* = − 12.55, β = − .23, SE = 13.13, *p* = .34) or men (*B* = 10.19, β = 0.20, SE = 13.66, *p* = .46). However, the testosterone by APOE4 interaction on p-Tau in the overall sample appeared to be mostly driven by women in that the testosterone and p-Tau relationship was marginally significant among female APOE4 carriers (*B* = − 18.06, β = − 0.34, SE = 8.89, *p* = .05) but not among female non-carriers (*B* = − 0.27, β = − 0.01, SE = 5.1, *p* = .96; Fig. 3). In contrast, the testosterone and p-Tau relationship was a trend in both male APOE4 carriers (*B* = − 13.38, β = − 0.26, SE = 6.75, *p* = .053) and non-carriers (*B* = − 24.24, β = − 0.26, SE = 13.78, *p* = .08), Despite the specificity of a testosterone and p-Tau link to female APOE4 carriers, we were likely underpowered to detect a APOE4 by testosterone interaction given the smaller sample size in female-specific analyses (*n* = 53). Results in both men and women were unchanged when substituting free for total testosterone and when adjusting for Aβ levels.

### Explanatory role of testosterone in sex difference in p-Tau

In testing the mediating role of testosterone in the sex difference in p-Tau levels, we found that the significantly higher p-Tau levels in female APOE4 carriers versus male APOE4 carriers was eliminated after adjusting for testosterone levels (*B* = 3.21, β = 0.09, SE = 5.78, *p* = .58; Fig. 1). Conversely, the trend for higher p-Tau levels in men versus women among APOE4 non-carriers changed minimally after adjusting for testosterone (*B* = 9.47, β = 0.34, SE = 5.84, *p* = .10). Again, results were unchanged when substituting free for total testosterone and when adjusting for Aβ levels.

## Discussion

In replication of previous findings, we found higher CSF p-Tau levels in women versus men specifically among APOE4 carriers. Our novel finding was significant relationship between low testosterone levels and higher p-Tau among APOE4 carriers. Our hypothesis concerning a potential mechanistic role of testosterone in the sex difference in p-Tau was supported in that the significant sex difference in p-Tau levels among APOE4 carriers was eliminated when adjusting for testosterone levels. Findings suggest that the lower testosterone levels in women are a significant contributor to their higher levels of p-Tau compared to men. Previous animal and cell culture studies have described a protective role of testosterone against Tau pathology [15, 16, 43]; however, to the best of our knowledge, we are the first to report a testosterone and Tau link in a human sample.

Testosterone offers a number of neuroprotective effects including improvements in synaptic plasticity [44, 45] and synaptic density in hippocampal neurons [46–49], heightened cerebral blood flow and glucose metabolism [50], reductions in inflammation and oxidative stress [51, 52], and prevention against Aβ plaque deposition and their neurotoxic effects [14, 53, 54]. Although the biological basis underlying the testosterone and tau link is unclear, most relevant to Tau pathogenesis may be testosterone’s anti-inflammatory actions [55]. A role for gliosis and neuroinflammation in Tauopathy is evidenced by greater microglial activity and altered inflammatory pathway markers (e.g., interleukin-6, tumor necrosis factor-α) correlating with Tau burden [56–59] as well as inflammation-related AD risk factors that contribute to Tauopathy such the genetic factors of TREM2 [60] and APOE4 [61] and the environmental factors of traumatic brain injury [62, 63] and viral infection [64, 65]. Evidence suggests bidirectional effects between neuroinflammation and Tau propagation whereby inflammation can initiate and propagate Tau pathology while Tau aggregates can directly activate microglia and secretion of pro-inflammatory cytokines [66–68]. In early AD, Aβ plaques stimulate microgliosis and release of inflammatory cytokines [69] suggesting that testosterone’s protection against Aβ plaque deposition may contribute to its anti-inflammatory properties and, in turn, decreased p-Tau. However, our results were unchanged after adjusting for Aβ suggesting that the mechanisms underlying the testosterone and p-Tau link are independent of Aβ. Research into the potential mediating role of neuroinflammation in the testosterone and Tau link is warranted.

Prior studies have also reported a testosterone by APOE4 interaction on cognitive function in animal models [37, 38] and on AD risk [31] and hippocampal volume [39] in humans. Similar to the majority of these studies, the pattern of interactive effects indicated an association between testosterone and p-Tau only among APOE4 carriers. In fact, the inclusion of APOE4 non-carriers, particularly women, in our analyses across APOE4 status weakened the relationship observed between testosterone and p-Tau among APOE4 carriers. In the sex-stratified analyses, the testosterone and p-Tau relationship is stronger in men versus women when combining APOE4 carriers and non-carriers; however, this relationship is stronger in female versus male APOE4 carriers. These findings underscore the importance of accounting for APOE4 status when examining testosterone and tau links.

There is biological plausibility for a testosterone by APOE interaction. In the brain, the APOE protein is a key transporter of lipoproteins. Given testosterone’s role in triglyceride and high-density lipoprotein cholesterol metabolism [70, 71], the shared role of APOE and testosterone in this lipoprotein pathway offers possibilities for interaction. The APOE4 allele is associated with an increased susceptibility to inflammation [61]. Thus, it is possible that APOE4 carriers are the most likely to benefit from the testosterone’s protective actions against inflammation and, in turn, Tau. In animal studies, APOE4 is associated with a reduction in cytosolic androgen receptor (AR) levels in the neocortex [37] leading to the possibility that the adverse effects of low testosterone levels are further amplified in APOE4 carriers that have fewer or less efficient AR to support testosterone signaling. We extend pervious findings by demonstrating testosterone by APOE4 interactive effects on p-Tau and their potential specificity to women.

Our results suggest that higher levels of p-Tau in women versus men are likely capturing an association between the low testosterone levels that are commonly seen in women and higher p-Tau. In fact, we found that the higher p-Tau levels in female APOE4 carriers versus male APOE4 carriers was eliminated when adjusting for testosterone suggesting that differences in testosterone between men and women is a central mechanism underlying this sex difference. These findings may have implications for the well-evidenced higher AD risk in women considering that Tau pathology is closely tied to neurodegeneration and clinical symptomology. Our findings also challenge the concept that testosterone is a “male hormone” in which the implications of low levels on AD-related outcomes are mostly circumscribed to men despite women having lower testosterone levels than men overall as well as age-related declines.

Our results offer a potential mechanism for the strongly, yet not consistently [72] supported finding of a stronger effect of APOE4 in women versus men on AD risk [6–8, 72]. If APOE4 has a stronger effect on AD-related outcomes in the context of low testosterone levels, as suggested by our data, then this would lead to a greater susceptibility of women to these effects. Our findings may also help to explain inconsistencies in the literature regarding an effect of APOE4 on Tau. Other biomarker [73], neuroimaging [74], and autopsy [75] studies found a more robust association between APOE4 and Tau in women versus men, whereas studies that did not compare by sex have shown inconsistent findings in the APOE4 and Tau link [73, 76–80]. If the Tau and APOE4 relationship is dependent on testosterone, as our results suggest, the presence of this relationship may be related to the proportion of men versus women in a sample. In non-sex-stratified analyses, an association between APOE4 and Tau may be obscured in samples that are predominantly male and, thus, likely characterized by higher testosterone levels.

This study has limitations. Our smaller sample size likely limited statistical power particularly when examining the testosterone and APOE4 interaction in sex-stratified analyses. Levels of circulating estradiol were not available in the ADNI, which precluded us from examining whether it is testosterone or the aromatization of testosterone to estradiol that is responsible for the observed association. However, previous animal work found testosterone’s neuroprotective effects against Aβ [81] and p-Tau [16] to be independent of estradiol levels suggesting that androgenic mechanisms are implicated in these effects [81]. CSF levels of testosterone may be more reflective of testosterone activity in the brain; however, only plasma-based levels were available to us. Because of our cross-sectional design, we were precluded from determining the temporal relationship between testosterone and Tau. Although previous findings suggest that testosterone’s effects predate AD outcomes, there is potential for bidirectional given evidence that AD pathology may negatively feedback on testosterone levels by hindering production of sex steroid hormones [82, 83]. Lastly, ADNI is a convenience sample of mostly white and well-educated volunteers compared with the general US population, which limits generalizability of results.

In conclusion, we found a relationship between lower testosterone levels and higher CSF p-Tau that was specific to APOE4 carriers. The specificity of this relationship to APOE4 carriers seemed to be driven by women. We replicated a consistent finding of higher p-Tau levels in women versus men at-risk for AD; however, this difference was eliminated after adjusting for testosterone. Results suggest that testosterone has a protective role against Tau particularly among APOE4 carriers, and that low testosterone levels that are more characteristic of women than men may predispose one to Tau.

### Perspectives and significance

Our findings inform a knowledge gap in our understanding of greater Tauopathy in women versus men on the AD trajectory and in the repeated demonstration of a stronger APOE4 effect in women. Our findings may also help to enlighten disparities in the literature regarding an APOE4 and Tau relationship. This study represents a call to researchers and clinicians that it is equally important to examine the effects of testosterone on AD-related outcomes in women as it is in men, if not more. Our findings stress the need to examine the effects of testosterone on AD-related outcomes in women in addition to men. Our findings have clinical relevance in that low testosterone is a potentially modifiable risk factor. Although numerous studies have investigated the effects of testosterone supplementation on cognitive function and AD risk with mixed findings (Wolf et al. 1999), very few studies have examined the effects of testosterone supplementation in women and with regard to APOE4 status. Follow-up studies should investigate (a) the association between testosterone levels and cortical Tau as measured by PET, (b) the effect of testosterone supplementation on Tau burden, and (c) the mediating role of neuroinflammation in the testosterone and Tau link.

## Data Availability

The dataset supporting the conclusions of this article is available in the ADNI repository [adni.loni.usc.edu].

## References

[1] Andersen K, Launer LJ, Dewey ME, Letenneur L, Ott A, Copeland JRM (1999). Gender differences in the incidence of AD and vascular dementia: The EURODEM Studies. Neurology..

[2] Jorm AF, Korten AE, Henderson AS (1987). The prevalence of dementia: a quantitative integration of the literature. Acta Psychiatr Scand..

[3] Lin KA, Choudhury KR, Rathakrishnan BG, Marks DM, Petrella JR, Doraiswamy PM (2015). Marked gender differences in progression of mild cognitive impairment over 8 years. Alzheimer’s Dement Transl Res Clin Interv..

[4] Sundermann EE, Biegon A, Rubin LH, Lipton RB, Mowrey W, Landau S (2016). Better verbal memory in women than men in MCI despite similar levels of hippocampal atrophy. Neurology..

[5] Sundermann EE, Biegon A, Rubin LH, Lipton RB, Landau S, Maki PM (2017). Does the female advantage in verbal memory contribute to underestimating Alzheimer’s disease pathology in women versus men?. J Alzheimer’s Dis..

[6] Bretsky PM, Buckwalter JG, Seeman TE, Miller CA, Poirier J, Schellenberg GD (1999). Evidence for an interaction between apolipoprotein E genotype, gender, and Alzheimer disease. Alzheimer Dis Assoc Disord..

[7] Payami H, Zareparsi S, Montee KR, Sexton GJ, Kaye JA, Bird TD (1996). Gender difference in apolipoprotein E - associated risk for familial alzheimer disease: A possible clue to the higher incidence of alzheimer disease in women. Am J Hum Genet..

[8] Poirier J, Bertrand P, Poirier J, Kogan S, Gauthier S, Poirier J (1993). Apolipoprotein E polymorphism and Alzheimer’s disease. Lancet..

[9] Oveisgharan S, Arvanitakis Z, Yu L, Farfel J, Schneider JA, Bennett DA (2018). Sex differences in Alzheimer’s disease and common neuropathologies of aging. Acta Neuropathol..

[10] Buckley RF, Mormino EC, Rabin JS, Hohman TJ, Landau S, Hanseeuw BJ (2019). Sex differences in the Association of Global Amyloid and Regional Tau Deposition measured by positron emission tomography in clinically normal older adults. JAMA Neurol..

[11] Hohman TJ, Dumitrescu L, Barnes LL, Thambisetty M, Beecham G, Kunkle B (2018). Sex-specific association of apolipoprotein e with cerebrospinal fluid levels of tau. JAMA Neurol..

[12] Altmann A, Tian L, Henderson VW, Greicius MD (2014). Sex modifies the APOE-related risk of developing Alzheimer disease. Ann Neurol..

[13] Ossenkoppele R, Schonhaut DR, Schöll M, Lockhart SN, Ayakta N, Baker SL (2016). Tau PET patterns mirror clinical and neuroanatomical variability in Alzheimer’s disease. Brain..

[14] Rosario ER, Carroll J, Pike CJ (2010). Testosterone regulation of Alzheimer-like neuropathology in male 3xTg-AD mice involves both estrogen and androgen pathways. Brain Res..

[15] Papasozomenos SC (1997). The heat shock-induced hyperphosphorylation of τ is estrogen- independent and prevented by androgens: Implications for Alzheimer disease. Proc Natl Acad Sci U S A..

[16] Papasozomenos SC, Shanavas A (2002). Testosterone prevents the heat shock-induced overactivation of glycogen synthase kinase-3β but not of cyclin-dependent kinase 5 and c-Jun NH2-terminal kinase and concomitantly abolishes hyperphosphorylation of τ: Implications for Alzheimer’s disease. Proc Natl Acad Sci U S A..

[17] Davison SL, Davis SR (2003). Androgens in women. J Steroid Biochem Mol Biol..

[18] Pike CJ, Carroll JC, Rosario ER, Barron AM (2009). Protective actions of sex steroid hormones in Alzheimer’s disease. Front. Neuroendocrinol..

[19] J. Wahjoepramono E, R. Asih P, Aniwiyanti V, Taddei K, S. Dhaliwal S, J. Fuller S, et al. The effects of testosterone supplementation on cognitive functioning in older men. CNS Neurol Disord - Drug Targets. 2016;15(3):337-343. 10.2174/1871527315666151110125704 PMC5078598 26553159

[20] Verdile G, Laws SM, Henley D, Ames D, Bush AI, Ellis KA (2014). Associations between gonadotropins, testosterone and β amyloid in men at risk of Alzheimer’s disease. Mol Psychiatry..

[21] Hogervorst E, Combrinck M, Smith AD (2003). Testosterone and gonadotropin levels in men with dementia. Neuroendocrinol Lett..

[22] Hogervorst E, Williams J, Budge M, Barnetson L, Combrinck M, Smith AD (2001). Serum total testosterone is lower in men with Alzheimer’s disease. Neuroendocrinol Lett..

[23] Moffat SD, Zonderman AB, Metter EJ, Kawas C, Blackman MR, Harman SM (2004). Free testosterone and risk for Alzheimer disease in older men. Neurology..

[24] Geerlings MI, Strozyk D, Masaki K, Remaley AT, Petrovitch H, Ross GW (2006). Endogenous sex hormones, cognitive decline, and future dementia in old men. Ann Neurol..

[25] Leblanc ES, Wang PY, Janowsky JS, Neiss MB, Fink HA, Yaffe K (2010). Association between sex steroids and cognition in elderly men. Clin Endocrinol (Oxf)..

[26] Hogervorst E, Matthews FE, Brayne C (2010). Are optimal levels of testosterone associated with better cognitive function in healthy older women and men?. Biochim Biophys Acta - Gen Subj..

[27] Thilers PP, MacDonald SWS, Herlitz A (2006). The association between endogenous free testosterone and cognitive performance: A population-based study in 35 to 90 year-oldmen and women. Psychoneuroendocrinology..

[28] Yaffe K, Lui LY, Zmuda J, Cauley J (2002). Sex hormones and cognitive function in older men. J Am Geriatr Soc..

[29] Barrett-Connor E, Goodman-Gruen D, Patay B (1999). Endogenous sex hormones and cognitive function in older men 1. J Clin Endocrinol Metab..

[30] Boss L, Kang DH, Marcus M, Bergstrom N. Endogenous sex hormones and cognitive function in older adults: a systematic review. West. J. Nurs. Res. 2014. 10.1177/0193945913500566 23996907

[31] Hogervorst E, Lehmann DJ, Warden DR, McBroom J, Smith AD (2002). Apolipoprotein E ε4 and testosterone interact in the risk of Alzheimer’s disease in men. Int J Geriatr Psychiatry..

[32] Chu L, Tam S, Lee PWH, Yik P-Y, Song Y, Cheung BMY (2010). Bioavailable testosterone decreases the risk of Alzheimer’s disease in older men. Alzheimer’s Dement..

[33] Tan S, Sohrabi HR, Weinborn M, Tegg M, Bucks RS, Taddei K (2019). Effects of Testosterone Supplementation on Separate Cognitive Domains in Cognitively Healthy Older Men: A Meta-analysis of Current Randomized Clinical Trials. Am. J. Geriatr. Psychiatry..

[34] Cherrier MM, Asthana S, Plymate S, Baker L, Matsumoto AM, Peskind E (2001). Testosterone supplementation improves spatial and verbal memory in healthy older men. Neurology..

[35] Cherrier MM, Matsumoto AM, Amory JK, Asthana S, Bremner W, Peskind ER (2005). Testosterone improves spatial memory in men with Alzheimer disease and mild cognitive impairment. Neurology..

[36] Resnick SM, Matsumoto AM, Stephens-Shields AJ, Ellenberg SS, Gill TM, Shumaker SA (2017). Testosterone treatment and cognitive function in older men with low testosterone and age-associated memory impairment. JAMA - J Am Med Assoc..

[37] Raber J, Bongers G, LeFevour A, Buttini M, Mucke L (2002). Androgens protect against apolipoprotein E4-induced cognitive deficits. J Neurosci..

[38] Pfankuch T, Rizk A, Olsen R, Poage C, Raber J (2005). Role of circulating androgen levels in effects of apoE4 on cognitive function. Brain Res..

[39] Panizzon MS, Hauger R, Dale AM, Eaves LJ, Eyler LT, Fischl B (2010). Testosterone modifies the effect of APOE genotype on hippocampal volume in middle-aged men. Neurology..

[40] Panizzon MS, Hauger R, Xian H, Vuoksimaa E, Spoon KM, Mendoza SP, et al. Interaction of APOE genotype and testosterone on episodic memory in middle-aged men. Neurobiol Aging. 2014;35(7):1778:e1-8. 10.1016/j.neurobiolaging.2013.12.025 PMC3980008 24444806

[41] Hardy JA, Higgins GA (1992). Alzheimer’s disease: The amyloid cascade hypothesis. Science..

[42] Petersen RC, Aisen PS, Beckett LA, Donohue MC, Gamst AC, Harvey DJ (2010). Alzheimer’s disease neuroimaging initiative (ADNI): clinical characterization. Neurology..

[43] Papasozomenos SC, Papasozomenos T (1999). Androgens prevent the heat shock-induced hyperphosphorylation but not dephosphorylation of τ in female rats. Implications for Alzheimer’s disease. J Alzheimer’s Dis..

[44] Schulz K, Korz V (2010). Hippocampal testosterone relates to reference memory performance and synaptic plasticity in male rats. Front Behav Neurosci..

[45] Hajszan T, MacLusky NJ, Leranth C (2008). Role of androgens and the androgen receptor in remodeling of spine synapses in limbic brain areas. Horm. Behav..

[46] Leranth C, Hajszan T, MacLusky NJ (2004). Androgens Increase Spine Synapse Density in the CA1 Hippocampal Subfield of Ovariectomized Female Rats. J Neurosci..

[47] Jia J (2016). xin, Cui C li, Yan X sheng, Zhang B feng, Song W, Huo D sheng, et al. Effects of testosterone on synaptic plasticity mediated by androgen receptors in male SAMP8 mice. J Toxicol Environ Heal - Part A Curr Issues..

[48] Hamson DK, Wainwright SR, Taylor JR, Jones BA, Watson NV, Galea LAM (2013). Androgens increase survival of adult-born neurons in the dentate gyrus by an androgen receptor-dependent mechanism in male rats. Endocrinology..

[49] Leranth C, Petnehazy O, MacLusky NJ (2003). Gonadal hormones affect spine synaptic density in the CA1 hippocampal subfield of male rats. J Neurosci..

[50] Moffat SD, Resnick SM (2007). Long-term measures of free testosterone predict regional cerebral blood flow patterns in elderly men. Neurobiol Aging..

[51] Ahlbom E, Prins GS, Ceccatelli S (2001). Testosterone protects cerebellar granule cells from oxidative stress-induced cell death through a receptor mediated mechanism. Brain Res..

[52] Grimm A, Biliouris EE, Lang UE, Götz J, Mensah-Nyagan AG, Eckert A (2016). Sex hormone-related neurosteroids differentially rescue bioenergetic deficits induced by amyloid-β or hyperphosphorylated tau protein. Cell Mol Life Sci..

[53] Rosario ER, Pike CJ (2008). Androgen regulation of β-amyloid protein and the risk of Alzheimer’s disease. Brain Res. Rev..

[54] Ramsden M, Nyborg AC, Murphy MP, Chang L, Stanczyk FZ, Golde TE (2003). Androgens modulate β-amyloid levels in male rat brain. J Neurochem..

[55] Ota H, Akishita M, Akiyoshi T, Kahyo T, Setou M, Ogawa S (2012). Testosterone deficiency accelerates neuronal and vascular aging of samp8 mice: Protective role of enos and sirt1. PLoS One..

[56] Cras P, Kawai M, Siedlak S, Perry G (1991). Microglia are associated with the extracellular neurofibrillary tangles of alzheimer disease. Brain Res..

[57] Dipatre PL, Gelman BB (1997). Microglial cell activation in aging and Alzheimer disease: partial linkage with neurofibrillary tangle burden in the hippocampus. J Neuropathol Exp Neurol..

[58] Probst A, Ulrich J, Heitz PU (1982). Senile dementia of Alzheimer type: astroglial reaction to extracellular neurofibrillary tangles in the hippocampus - An immunocytochemical and electron-microscopic study. Acta Neuropathol..

[59] Sheffield LG, Marquis JG, Berman NEJ (2000). Regional distribution of cortical microglia parallels that of neurofibrillary tangles in Alzheimer’s disease. Neurosci Lett..

[60] Ulrich JD, Holtzman DM (2016). TREM2 Function in Alzheimer’s Disease and Neurodegeneration. ACS Chem. Neurosci..

[61] Ohm TG, Kirca M, Bohl J, Scharnagl H, Groß W, März W (1995). Apolipoprotein E polymorphism influences not only cerebral senile plaque load but also Alzheimer-type neurofibrillary tangle formation. Neuroscience..

[62] McKee AC, Cairns NJ, Dickson DW, Folkerth RD, Dirk Keene C, Litvan I (2016). The first NINDS/NIBIB consensus meeting to define neuropathological criteria for the diagnosis of chronic traumatic encephalopathy. Acta Neuropathol..

[63] Cherry JD, Tripodis Y, Alvarez VE, Huber B, Kiernan PT, Daneshvar DH (2016). Microglial neuroinflammation contributes to tau accumulation in chronic traumatic encephalopathy. Acta Neuropathol Commun..

[64] Cannon JR, Greenamyre JT (2011). The role of environmental exposures in neurodegeneration and neurodegenerative diseases. Toxicol Sci..

[65] Harris SA, Harris EA (2018). Molecular mechanisms for herpes simplex virus type 1 pathogenesis in Alzheimer’s disease. Front. Aging Neurosci..

[66] Eikelenboom P, Van Exel E, Hoozemans JJM, Veerhuis R, Rozemuller AJM, Van Gool WA (2010). Neuroinflammation - An early event in both the history and pathogenesis of Alzheimer’s disease. Neurodegener Dis..

[67] Laurent C, Buée L, Blum D (2018). Tau and neuroinflammation: what impact for Alzheimer’s disease and tauopathies?. Biomed. J..

[68] Vogels T, Murgoci A-N, Hromádka T (2019). Intersection of pathological tau and microglia at the synapse. Acta Neuropathol Commun..

[69] Wang WY, Tan MS, Yu JT, Tan L (2015). Role of pro-inflammatory cytokines released from microglia in Alzheimer’s disease. Ann. Transl. Med..

[70] Nordøy A, Aakvaag A, Thelle D (1979). Sex hormones and high density lipoproteins in healthy males. Atherosclerosis..

[71] Perova NV, Gerasimova EN, Chernysheva NP (1979). Change in the apoproteins of very low density lipoproteins in the blood in hypertriglyceridemia. Vopr Meditsinskoj Khimii..

[72] Neu SC, Pa J, Kukull W, Beekly D, Kuzma A, Gangadharan P (2017). Apolipoprotein E genotype and sex risk factors for Alzheimer disease: A meta-analysis. JAMA Neurol..

[73] Toledo JB, Zetterberg H, Van Harten AC, Glodzik L, Martinez-Lage P, Bocchio-Chiavetto L (2015). Alzheimer’s disease cerebrospinal fluid biomarker in cognitively normal subjects. Brain..

[74] Liu M, Paranjpe MD, Zhou X, Duy PQ, Goyal MS, Benzinger TLS (2019). Sex modulates the ApoE ε4 effect on brain tau deposition measured by 18F-AV-1451 PET in individuals with mild cognitive impairment. Theranostics..

[75] Corder EH, Ghebremedhin E, Taylor MG, Thal DR, Ohm TG, Braak H (2004). The biphasic relationship between regional brain senile plaque and neurofibrillary tangle distributions: Modification by age, sex, and APOE polymorphism. Ann N Y Acad Sci..

[76] Lautner R, Palmqvist S, Mattsson N, Andreasson U, Wallin A, Pålsson E (2014). Apolipoprotein e genotype and the diagnostic accuracy of cerebrospinal fluid biomarkers for alzheimer disease. JAMA Psychiatry..

[77] Sunderland T, Mirza N, Putnam KT, Linker G, Bhupali D, Durham R (2004). Cerebrospinal fluid β-amyloid 1-42 and tau in control subjects at risk for Alzheimer’s disease: the effect of APOE ε4 allele. Biol Psychiatry..

[78] Vemuri P, Wiste HJ, Weigand SD, Knopman DS, Shaw LM, Trojanowski JQ (2010). Effect of apolipoprotein E on biomarkers of amyloid load and neuronal pathology in Alzheimer disease. Ann Neurol..

[79] Farfel JM, Yu L, De Jager PL, Schneider JA, Bennett DA (2016). Association of APOE with tau-tangle pathology with and without β-amyloid. Neurobiol Aging..

[80] Liu Y, Tan L, Wang HF, Liu Y, Hao XK, Tan CC (2016). Multiple effect of APOE genotype on clinical and neuroimaging biomarkers across Alzheimer’s Disease spectrum. Mol Neurobiol..

[81] Pike CJ (2001). Testosterone attenuates β-amyloid toxicity in cultured hippocampal neurons. Brain Res..

[82] Schaeffer V, Meyer L, Patte-Mensah C, Eckert A, Mensah-Nyagan AG (2008). Dose-dependent and sequence-sensitive effects of amyloid-β peptide on neurosteroidogenesis in human neuroblastoma cells. Neurochem Int..

[83] Schaeffer V, Patte-Mensah C, Eckert A, Mensah-Nyagan AG (2006). Modulation of neurosteroid production in human neuroblastoma cells by Alzheimer’s disease key proteins. J Neurobiol..

